# A Rare Presentation of Autonomously Functioning Papillary Thyroid Cancer: Malignancy in Marine-Lenhart Syndrome Nodule

**DOI:** 10.1155/2016/8740405

**Published:** 2016-03-27

**Authors:** Mehmet Uludag, Nurcihan Aygun, Alper Ozel, Feyza Yener Ozturk, Rabia Karasu, Banu Yilmaz Ozguven, Bulent Citgez, Mehmet Mihmanli, Adnan Isgor

**Affiliations:** ^1^Department of General Surgery, Sisli Hamidiye Etfal Training and Research Hospital, 34371 Istanbul, Turkey; ^2^Department of Radiology, Sisli Hamidiye Etfal Training and Research Hospital, 34371 Istanbul, Turkey; ^3^Department of Endocrinology and Metabolism, Sisli Hamidiye Etfal Training and Research Hospital, 34371 Istanbul, Turkey; ^4^Department of Pathology, Sisli Hamidiye Etfal Training and Research Hospital, 34371 Istanbul, Turkey; ^5^Department of General Surgery, Bahcesehir University Medical Faculty, 34353 Istanbul, Turkey

## Abstract

*Objective*. Marine-Lenhart Syndrome (MLS) is defined as concomitant occurrence of autonomously functioning thyroid nodule (AFTN) with Graves' disease (GD). Malignancy in a functional nodule is rare. We aimed to present an extremely rare case of papillary thyroid cancer in a MLS nodule with lateral lymph node metastases.* Case*. A 43-year-old male presented with hyperthyroidism and Graves' ophthalmopathy. On Tc99m pertechnetate scintigraphy, a hyperactive nodule in the left upper thyroid pole was detected and the remaining tissue showed a mildly increased uptake. The ultrasonography demonstrated 15.5 × 13.5 × 12 mm sized hypoechoic nodule in the left upper pole of the thyroid and round lymph nodes on the left side of the neck. Fine needle aspiration biopsy (FNAB) of the nodule and lymph node revealed cytological findings consistent with papillary cancer. Total thyroidectomy with central and left modified radical neck dissection was performed. On pathologic examination, two foci of micropapillary cancer were detected. The skip metastases were present in three lymph nodes on the neck.* Conclusion*. AFTN can be seen rarely in association with GD. It is not possible to exclude malignancy due to the clinical and imaging findings. In the presence of suspicious clinical and sonographic features, FNAB should be performed.

## 1. Introduction

Hyperthyroidism is a common disorder throughout the world. Graves' disease (GD) and autonomously functioning thyroid nodules (AFTN) are the most common causes of hyperthyroidism [1]. These two diseases have different pathophysiological mechanisms and clinical findings [2]. The coexistence of both diseases has been called Marine-Lenhart Syndrome (MLS) and first published by Marine and Lenhart in 1911 [3].

Thyroid nodules in patients with GD are common. The vast majority of these nodules are nonfunctioning. The presence of AFTN within a Graves' thyroid is rare, occurring in 1% to 2.7% of cases of GD [4, 5]. In general, the vast majority of AFTN are benign. A literature review of surgical case series with solitary hyperfunctioning thyroid nodules managed by thyroid resection revealed an estimated 3.1% prevalence of malignancy [6]. However, autonomously functioning differentiated thyroid cancers are reported as primary sporadic cases in the literature until 2013 [7].

We aimed to present an extremely rare case of a papillary cancer embedded in an AFTN of MLS, having clinical lymph node metastases.

## 2. Case Report

A 43-year-old male patient was admitted with the complaints of sweating, palpitation, tremor, eyes becoming remarkable, and weight loss for three months. His blood pressure was BP: 120/80 mmHg and heart rate 120 bpm. On physical examination, his thyroid was found to be moderately enlarged and a hard nodule having a size of 1.5 cm was palpable in the upper pole of the left lobe. A hard lymph node in size of 1 cm was palpable in the left lateral compartment (Level 3). Mild proptosis of the eyes and ptosis of the left eyelid were observed.

**Figure 1:**
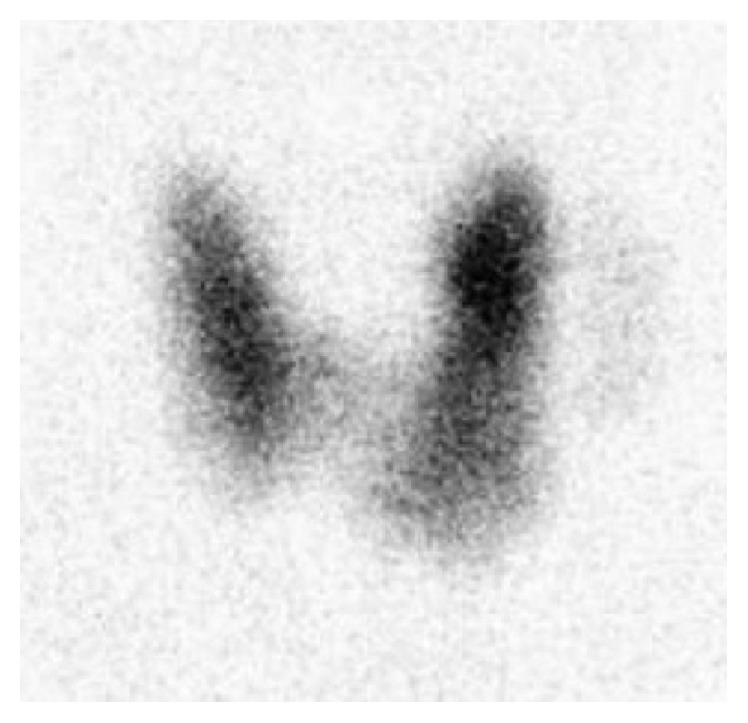
Anterior views of technetium 99m scan demonstrate a normal size, v-shaped thyroid gland with a hot area in the left upper pole consistent with hyperfunctioning nodule. The remaining tissue showed a slightly increased uptake but not increased as expected in GD.

Thyroid function tests revealed hyperthyroidism with elevated free triiodothyronine (FT3) of 5.68 pg/mL (normal: 1.64–4.42 pg/mL), free thyroxine (FT4) of 2.14 ng/dL (normal: 0.80–1.67 ng/dL), and suppressed thyroid-stimulating hormone (TSH) of <0.01 *μ*IU/mL (normal: 0.27–4.20 *μ*IU/mL). Thyroid peroxidase and thyroglobulin antibody levels were negative, but TSH receptor antibody was found to be 13 U/L, at the limit of being positive (normal: 9–14 U/L). Anterior views of technetium 99m scan demonstrate a normal size, v-shaped thyroid gland with a hot area in the left upper pole consistent with hyperfunctioning nodule. The remaining tissue showed a slightly increased uptake, but not increased as expected in GD (Figure 1).

**Figure 2:**
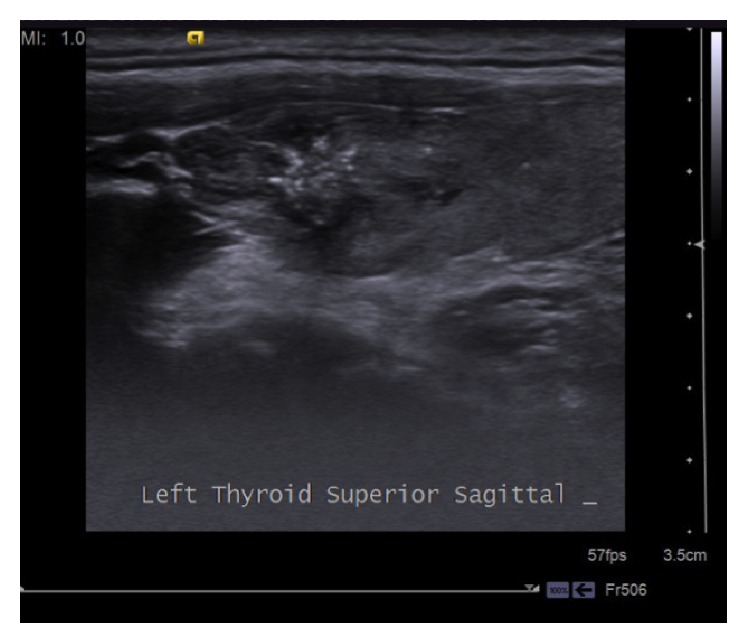
Thyroid ultrasound disclosed a hypoechoic nodule of 15.5 × 13.5 × 12 mm in size in the left upper pole, having irregular spiculated margins, and dense millimetric microcalcifications in the central area.

**Figure 3:**
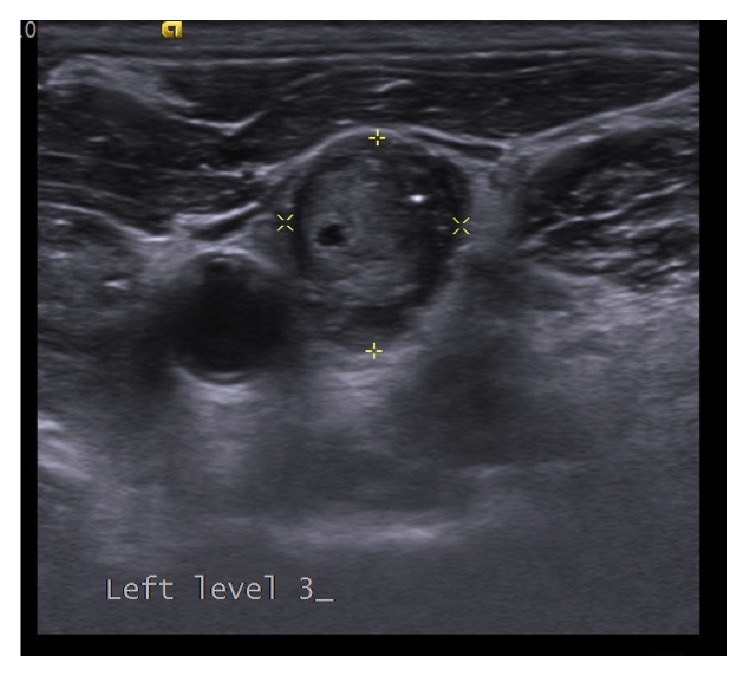
Transverse sonogram of left lateral neck demonstrates a 12 × 10 mm sized, rounded lymph node with internal cystic component and microcalcifications. The hilum of the lymph node could not be distinguished.

Thyroid ultrasound disclosed a hypoechoic nodule of 15.5 × 13.5 × 12 mm in size in the left upper pole, having irregular spiculated margins, dense millimetric microcalcifications in the central area (Figure 2), and vascularization both internally and externally. Ultrasound also showed several lymph nodes, the largest one being 12 × 10 mm in size with heterogeneous cortex, that are round, having multiple microcalcifications with cystic components and undistinguishable echogenic hilum, in Level 3 of left cervical region (Figure 3).

**Figure 4:**
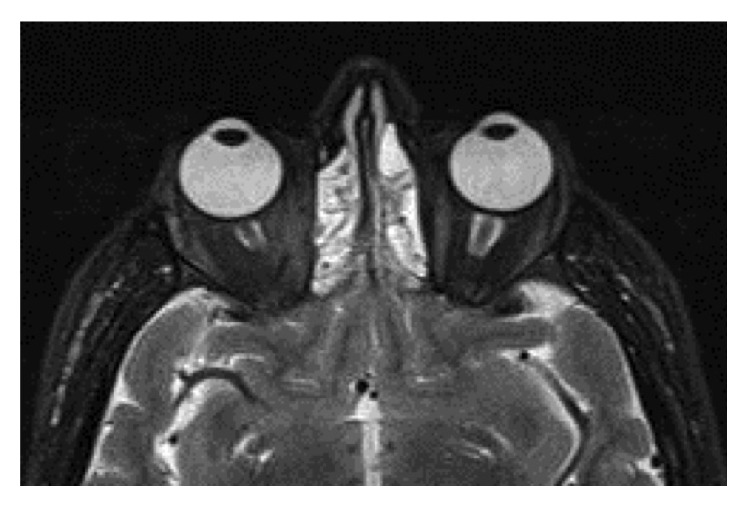
Orbital magnetic resonance imaging showed exophthalmos with enlargement of both eye muscles specially on the right.

**Figure 5:**
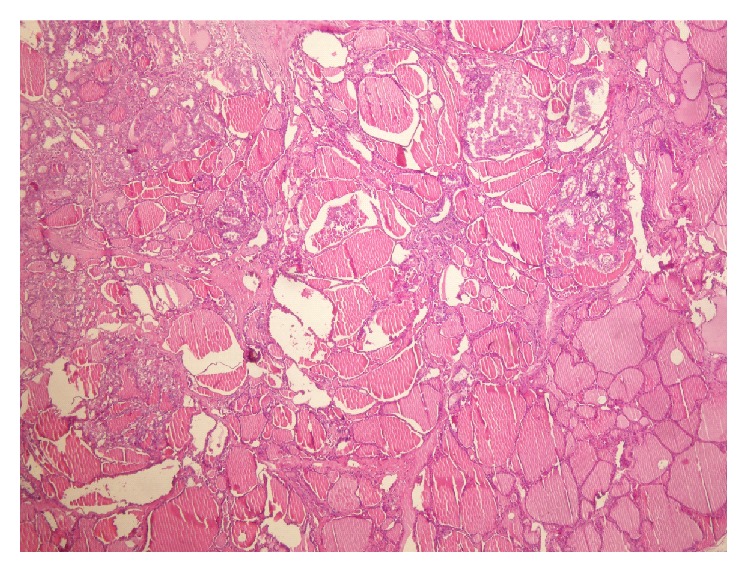
Papillary microcarcinoma focus developed in the adenomatous hyperplasia, H&E, ×40.

An ultrasound guided fine needle aspiration biopsy of the nodule and cervical lymph node, which showed suspicious sonographic features, was performed and both revealed papillary thyroid carcinoma. Thyroglobulin washout of fine needle aspirate from the lymphadenopathy was measured to be over 30000 ng/dL. Orbital magnetic resonance imaging showed exophthalmos with enlargement of the both eye muscles especially on the right (Figure 4). The patient was symptomatically and biochemically euthyroid on 20 mg/day dose of methimazole therapy after 4 weeks. Total thyroidectomy with central and left sided modified radical neck dissection was performed. Pathology disclosed two foci of papillary thyroid cancer, which were 3 mm and 8 mm in size, in the hyperfunctioning nodule localised in the left upper pole (Figure 5). Lymphovascular invasion was determinate. Encapsulation and extrathyroidal extension were not seen. The rest of the nodule is consisted of adenomatous hyperplasia. The rest of the gland revealed somewhat hyperfunctional changes of follicles. No metastasis was identified in the 11 lymph nodes dissected from the central neck region. Metastases were identified in 3 of 29 lymph nodes dissected from the lateral neck region, two of them being in Level 3 and one in Level 4. BRAF mutation test is negative to the carcinoma sections. The patient was given a treatment dose of 150 mCi of radioiodine I-131 postoperatively. Whole body scan was performed showing only focal I-131 uptake in the thyroidectomy bed, but not in any other parts of the body, on the 7th day posttherapy.

## 3. Discussion

Thyroid nodules in patients with GD are common, though the frequency varies depending on the method used [4]. Palpable thyroid nodules occur in approximately 15% of patients with GD compared with the usual rate of 5% observed in the general population [8]. When only clinical examination and scan are employed, the prevalence can be much lower as compared to when ultrasonography is used [9]. Kraimps et al. [9] reported a multicentric study including 557 cases in which 40 (28.6%) of 140 patients (prevalence 25.1%) with thyroid nodules could be detected by palpation. Scintigraphy disclosed 54 patients (38.6%) with cold nodules, whereas thyroid ultrasound detected 116 (82.9%) of these patients.

Although thyroid nodules are frequently present in GD, it is rare for these nodules to be functioning. Marine-Lenhart found 3 (4.3%) functioning nodules within 69 patients having GD in their original study. Charkes [5] detected 10 patients (2.7%) having incidental AFTN out of 375 patients with GD referred to a Nuclear Medicine Center for radioiodine testing. Carnell and Valente [4] detected 60 (12.8%) patients with thyroid nodules out of 468 Graves' patients. Sixty patients with nodules were classified as follows: a solitary hypofunctional nodule (*n* = 27, 5.8%); multiple nodules (*n* = 21, 4.5%); AFTN (*n* = 4, 1%); or patchy GD (*n* = 8, 1.7%). The rate of AFTNs is 1% in the total serial, but 6.7% in the patients with nodules. In another study, 35 (26.9%) patients had palpable nodules out of surgically treated 130 GD. Twenty-five of patients having palpable nodules performed scintigraphy, showing cold nodule in 15, warm in 1, and diffuse uptake in 9 cases. The frequency of simultaneous occurrence of Graves' ophthalmopathy and AFTNs is reported to be 0.05–0.2% [10]. A retrospective study, including three-thousand eight-hundred and thirty-nine consecutive patients, was made to analyze the ultrasonographic findings in scintigraphic hot areas. One hundred and four (2.7%) patients had scintigraphic hot areas. Only four of these patients were ophthalmic Graves' patients being euthyroid, having a rate of 3.8% in patients with hot area and 0.1% in total of the patients [11].

It is still controversial whether the frequency of thyroid cancer in patients with GD is higher or not. The incidence of malignancy in GD is reported to be 1.3–31.7% [5, 9, 12–17]. While Kraimps et al. [9] detected all thyroid carcinomas inside the nodules in their series, in other studies thyroid carcinoma was diagnosed in the parenchyma outside the nodules with the rates of 67% and 73.8% [14–16]. Nevertheless, the incidence of cancer in GD with nodules is higher than in GD without nodules [5, 9, 13, 14, 17]. The risk of malignancy is considerably increased in patients having GD with nodules [14, 17]. However, in a recent study the incidence of malignancy is reported to be similar in GD, toxic multinodular goiter, and nontoxic multinodular goiter [15].

The aggressiveness of thyroid cancer in GD is also a subject of controversy. Some studies have found that thyroid carcinomas in patients with GD grow more invasively, developing lymph node and distant metastases more frequently compared to patients with either AFTN or toxic multinodular goiter or euthyroid goiter [12, 18]. In another study, younger age is the only significant determined predictive factor for the development of lymph node metastases in Graves' patients with thyroid carcinoma [19]. Pellegriti et al. [8] have found in their last study that persistent/recurrent disease was more frequent in Graves' patients with differentiated thyroid cancer (DTC) compared to euthyroid control patients. They have also found that disease-specific mortality was significantly higher in Graves' patients with DTC (28.6%) compared to euthyroid control patients having DTC (2.9%) (*p* = 0.0001). They described that these findings point to the need for early diagnosis and also the importance of aggressive treatment of DTCs in patients having GD. In some other studies, these suggestions are not supported and it is defined that thyroid carcinoma in GD should be treated like the other patients having thyroid carcinoma [20, 21].

In the literature, autonomously functioning differentiated thyroid cancers are rare and reported as primary sporadic cases [7]. A MEDLINE literature search between 1950 and 2012, made by Mirfakhraee et al. [6] set out to determine the prevalence of malignant hot nodules, revealed 112 cases composed of 77 case reports and 35 cases out of surgical series.

The reported MLS cases in the literature are rare and usually benign [4, 5, 22]. The malignant cases that could be associated with MLS are extremely rare. We found five such cases in the literature [23–27].

In the first case, an autonomously functioning thyroid carcinoma in euthyroid Graves' patient with Graves' ophthalmopathy was reported. The Japanese patient underwent subtotal thyroidectomy and prophylactic right modified radical neck dissection, and a 1.5 × 1 cm sized papillary carcinoma and 9 microscopic lymph node carcinoma metastases were found out of dissected 22 lymph nodes [23].

In the second case, micropapillary thyroid cancer in a hot thyroid nodule and multilobar tumor foci in the thyroid parenchyma out of the hot thyroid nodule were found [24].

The reported third patient had 9 mm sized micropapillary thyroid cancer in a hyperfunctioning nodule. Additionally, the tumor was positively tested for BRAF V600 mutation [25].

In another case, thyroid cancer was detected in a different nodule out of hyperfunctioning toxic nodule in a patient with GD. In the patient followed up for GD, a 1.6 cm sized, regular contoured hypoechoic thyroid nodule was detected in a diffuse enlarged thyroid gland. One year later, an additional 1.1 cm sized irregular contoured second nodule was detected in the left lobe. Thyroid scintigraphy with I-131 demonstrated a hyperactive area localised in the larger nodule and a lower diffuse uptake in the remaining thyroid tissue. On the histopathologic evaluation of the thyroidectomy specimen, the larger nodule was a hyperfunctioning follicular adenoma that caused the patient diagnosed as MLS, and the smaller nodule was an unencapsulated papillary carcinoma with several other microfoci of papillary carcinoma in the adjacent tissue. The authors claimed that the chronic abnormal stimulation of the thyroid gland by the thyroid-stimulating antibody (TSAb) may facilitate the neoplastic transformation of the thyrocytes [26].

In the last and fifth case, a papillary thyroid carcinoma was detected in a 2.5 cm sized hyperfunctioning nodule [27].

To our knowledge, our case is the first case of papillary thyroid cancer in Marine-Lenhart Syndrome toxic nodule with clinical lymph node metastasis. Besides, it is the fifth case in the literature with papillary thyroid cancer in Marine-Lenhart Syndrome nodule.

Skip metastasis, referred to as leaping metastasis to the lateral neck without associated lymphadenopathy in the central compartment (Level VI), can occur in patients with papillary thyroid carcinoma. Our case is also the first case with malign nodule of the MLS in the literature, regarding the lateral skip metastasis peculiarity. Skip metastases occur in a minority of patients with papillary thyroid microcarcinoma. Chung et al. [28] reported 7.7% skip metastases in patients with the suspicion of metastasis evaluated preoperatively by ultrasonography or computed tomography. Skip metastases occurred commonly with primary tumors of the upper pole as in our case. Patients with skip metastases had fever metastatic lateral nodes that were more frequently found at a single level: mostly at Level III [29]. Although BRAF mutation is negative, the lymph node metastasis may be attributed to the aggressive behavior of the thyroid cancer in GD.

Since hyperfunctioning nodules rarely harbor malignancy, if one is found that corresponds to the nodule in question, no routine cytologic evaluation is necessary. However, it is reasonable to evaluate an AFTN that has clinical suspicion or sonographic features similar to our case, in hyperthyroid patients before radioactive iodine treatment or surgical procedure. It is also suggested in the literature that a nodule can be evaluated with FNAB in patients with hyperthyroidism, prior to iodine treatment or surgical intervention [30]. Sonographic features and malignancy potential of nontoxic autonomous nodules resemble toxic autonomous nodules. No significant difference was detected in terms of cytological diagnosis, between toxic and nontoxic autonomous nodules [31].

The hyperfunctioning state of a thyroid nodule does not exclude the possibility of malignancy definitely. Toxic nodule in MLS can also be malignant, although extremely rare. Therefore, careful physical examination and ultrasonographic evaluation should be performed in all patients, and in case of presence of clinically and sonographically suspicious nodules, fine needle aspiration biopsy should be performed even in the presence of hyperfunctionality. Additionally, fine needle aspiration biopsy positive hyperfunctioning nodules should be searched for lymph node metastasis.
